# Tissue proteomics outlines AGR2 AND LOX5 as markers for biochemical recurrence of prostate cancer

**DOI:** 10.18632/oncotarget.26342

**Published:** 2018-11-23

**Authors:** Giovanny Rodríguez-Blanco, Lona Zeneyedpour, Diederick Duijvesz, A. Marije Hoogland, Esther I. Verhoef, Charlotte F. Kweldam, Peter C. Burgers, Peter Sillevis Smitt, Chris H. Bangma, Guido Jenster, Geert J.L.H. van Leenders, Lennard J.M. Dekker, Theo M. Luider

**Affiliations:** ^1^ Department of Neurology, Erasmus Medical Centre, Rotterdam, The Netherlands; ^2^ Department of Urology, Erasmus Medical Centre, Rotterdam, The Netherlands; ^3^ Department of Pathology, Erasmus Medical Centre, Rotterdam, The Netherlands

**Keywords:** prostate cancer, mass spectrometry, arachidonic acid

## Abstract

Although many patients are cured from prostate cancer (PCa) by surgery only, there are still patients who will experience rising prostate-specific antigen (PSA) levels after surgery, a condition known as biochemical recurrence (BCR). Novel protein prognostic markers in PCa tissue might enable finding better treatment for those patients experiencing BCR with a high chance of metastasis. In this study, we aimed to identify altered proteins in prostate cancer tissue, and to evaluate their potential role as prognostic markers. We used two proteomics strategies to analyse 34 prostate tumours (PCa) and 33 normal adjacent prostate (NAP) tissues. An independent cohort of 481 samples was used to evaluate the expression of three proteins: AGR2, FASN and LOX5 as prognostic markers of the disease. Tissue microarray immunohistochemical staining indicated that a low percentage of positive tumour cells for AGR2 (HR (95% CI) = 0.61 (0.43-0.93)), and a low percentage of positive tumour cells for LOX5 expression (HR (95% CI) = 2.53 (1.23-5.22)) are predictors of BCR after RP. In contrast, FASN expression had no prognostic value for PCa.

## INTRODUCTION

Prostate cancer (PCa) remains to date the most commonly diagnosed cancer in men in the Western world [1]. Although many patients are cured from this disease after radical prostatectomy (RP) [2], one third of patients will show an increment in serum PSA levels -also known as biochemical recurrence (BCR)-[3]. For those patients, more frequent follow-up and adjuvant therapies are often required to limit progression of the disease [3, 4]. There is a high need for robust molecular markers that can distinguish indolent cases of PCa from those that will recur after initial treatment [3, 4].

Small molecules, such as metabolites and lipids, have been associated with the progression of different types of cancer, including PCa [5]. In our previous study, using a LC-MS/MS-based targeted metabolomics method, we found lower concentrations of arachidonic acid (AA) in serum from PCa patients in the most aggressive stage of the disease [6]. In addition, serum levels of hydroxyeicosatetraenoic acid (HETE) metabolites, which are produced by lipoxygenase-type enzymes from AA, were elevated in serum of part of the patients within the same group of advanced PCa [6]. At tissue level, it has been reported that levels of AA in PCa were significantly lower compared to benign prostate tissues [7]. In addition, Yang *et al*. analysed PCa core biopsies and they found that the 15-LOX-2 metabolite 15-HETE, was higher in PCa than in the normal cores [8]. These findings suggest that the AA pathway might play an important role in PCa development and progression. However, analysis of proteins of the AA pathway in PCa, as well as their role in the prognosis of PCa is still unknown. In this study, we used two complementary mass spectrometry approaches to identify differentially expressed proteins in PCa tissue that could be used as prognostic markers for this disease. Protein signatures were validated in an extended cohort using immunohistochemistry on archival PCa tissue and an available tissue microarray. The expression of the selected proteins was evaluated for prediction of biochemical recurrence after radical prostatectomy.

## RESULTS

### Proteomics

**Figure 1:**
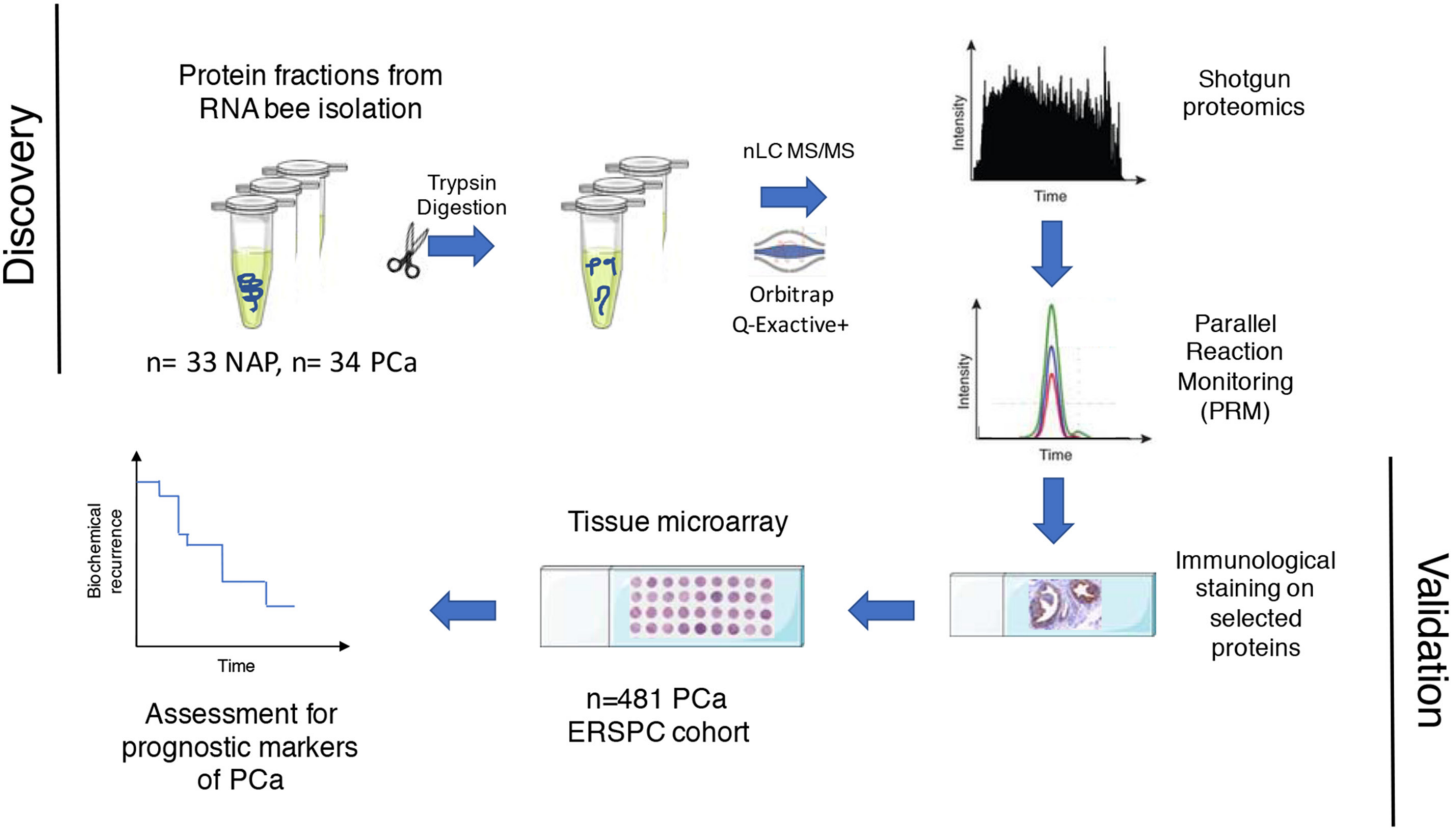
Workflow for finding proteins in PCa tissue that relate to prediction of biochemical recurrence

**Figure 2:**
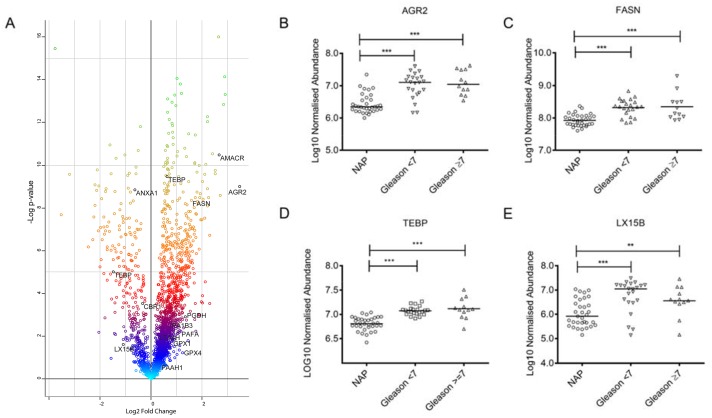
**(A)** Scatter plot of the normalized abundance mean ratio between PCa (n=34) and NAP (n=33) using the shotgun approach. Proteins belonging to the AA pathway arepresented in red (upregulated) and blue (down-regulated). **(B-E)** Boxplots of normalized abundances at different Gleason scores for proteins AGR2, FASN, TEBP and LX15B.

In this study we aimed to find protein signatures of PCa with potential applicability towards prognosis of the disease. To identify differentially expressed proteins in PCa, we used shotgun proteomics using the protein fraction from RNA-bee isolation of 34 PCa and 33 NAP tissues (Figure 1). Using label free quantification (LFQ), a total of 2865 proteins were identified, and 798 proteins were statistically significant (FDR<0.01). Figure 2A illustrates the LFQ mean ratio between PCa and NAP, also indicating that an elevated number of proteins were up-regulated in PCa. The list of identified proteins as well as the differentially expressed proteins in PCa is presented in Supplementary Table 2. Two proteins, anterior gradient 2, AGR2, and fatty acid synthase, FASN, were highly up-regulated in PCa and their normalised abundances showed also a trend when compared with the Gleason score, as shown in Figure 2B-2C. These two proteins were considered for further analysis.

To analyse whether proteins in the Arachidonic acid (AA) pathway could be used as prognostic markers for PCa, we manually searched the list of differentially expressed proteins and compared this list with the list of 79 proteins in the AA pathway as described by Sabidó *et al*. [9] (Supplementary Table 3). Interestingly, we identified fifteen proteins of the AA pathway in the list of de-regulated proteins, and particularly, prostaglandin E synthase 3. TEBP, showed a degree of correlation with the Gleason score, as is shown in Figure 2D.

We previously reported high levels of HETE metabolites in serum from PCa patients. In our shotgun proteomics dataset we only identified one lipoxygenase-type enzyme deregulated in PCa tissue: LX15B, but its expression is higher in NAP and the statistical test is significant (p<0.05), as shown in Figure 2E. The deregulated proteins in PCa which are part of the AA pathway are presented in Figure 2A.

Gleason score, pT Stage and ERG oncogene status have been associated with poor prognosis of PCa. To evaluate whether proteins identified in PCa tissue could be associated with these clinical parameters, we performed statistically tests on patients with different Gleason scores (GS 6 vs. GS 7), different pT stages (pT2, pT3, pT3) and whether ERG is activated or not (ERG oncogene-positive vs. ERG oncogene-negative). We did not find statistically significant proteins when comparing Gleason score or pT stage, but we found that 15 proteins were statistically significant when comparing ERG activation. Interestingly, three of these proteins belonging to the AA pathway and were up-regulated (FDR<0.01): phospholipase A2, PA2GA, arachidonate 15-lipoxygenase B, LX15B, and prostaglandin reductase 1, PTGR1. The list of differentially expressed proteins when ERG is activated is presented in Supplementary Table 4. These results indicate that the AA pathway might play a role in ERG activation.

To validate the results obtained in the shotgun proteomics experiment, we performed parallel reaction monitoring measurements (PRM) on the same samples described above, and we included other proteins not identified by shotgun approach in order to evaluate their potential as prognostic markers for PCa. We selected these proteins (Table 1) because they are involved in the metabolism of small molecules and fatty acids (polyamines, eicosanoids and phospholipids), as well as in the so-called Warburg effect, which also involves metabolic enzymes of metabolites present in the TCA cycle [10, 11]. In total we analysed 18 proteins and each protein was quantified using two peptides. We included two peptides for PARK7, a protein recently described for normalisation of proteomics experiments [12], and as expected, peak areas between samples were not statistically significant (p>0.05) different.

**Table 1:** List of transitions used in the Parallel Reaction Monitoring (PRM) experiments

Gene Name	Protein name and accession	Peptide	m/z	z	RT	RT	HCD Collision Energy (%)
					start (min)	stop (min)	
LOX5	**Arachidonate 5-lipoxygenase**	DDGLLVWEAIR	643.8406	2	46.13	48.13	27
	P09917 (LOX5_HUMAN)	GVVTIEQIVDTLPDR	827.9542	2	45.5	47.5	27
GLS	**Glutaminase**	GSTHPQPGVSPPAAPAAPGPK	641.0024	3	10.69	12.69	27
	O94925 (GLSK_HUMAN)	YAIAVNDLGTEYVHR	574.2933	3	25.63	27.63	27
ALOX15B	**Arachidonate 15-lipoxygenase B**	ELLIVPGQVVDR	669.393	2	31.3	33.3	27
	O15296 (LX15B_HUMAN)	STGIGIEGFSELIQR	803.9254	2	41.27	43.27	27
LTA4H	**Leukotriene A-4 hydrolase**	LTYTAEVSVPK	604.3321	2	18.73	20.73	27
	P09960 (LKHA4_HUMAN)	ELVALMSAIR	551.8181	2	33.83	35.83	27
PTGES3	**Prostaglandin E synthase 3**	LTFSC[+57.0]LGGSDNFK	723.3401	2	28.05	30.05	27
	Q15185 (TEBP_HUMAN)	LNWLSVDFNNWK	768.3857	2	47.25	49.25	27
EPHX2	**Bifunctional epoxide hydrolase 2**	FLLDTLK	425.2577	2	27.61	29.61	27
	P34913 (HYES_HUMAN)	AVASLNTPFIPANPNMSPLESIK	804.4262	3	43.54	45.54	27
FAAH	**Fatty-acid amide hydrolase 1**	LQNPDLDSEALLALPLPQLVQK	805.788	3	52.9	54.9	27
	O00519 (FAAH1_HUMAN)	ELAPEAVLFTYVGK	768.919	2	44.96	46.96	27
ACSL3	**Long-chain-fatty-acid–CoA ligase 3**	LQAGEYVSLGK	582.8166	2	18.53	20.53	27
	O95573 (ACSL3_HUMAN)	VLSEAAISASLEK	659.3666	2	24.13	26.13	27
SRM	**Spermidine synthase**	LTLHVGDGFEFMK	498.5868	3	32.79	34.79	27
	19623 (SPEE_HUMAN)	ESYYQLMK	531.2522	2	19.26	21.26	27
PRMT1	**Protein arginine N-methyltransferase 1**	ATLYVTAIEDR	626.3326	2	23.36	25.36	27
	Q99873 (ANM1_HUMAN)	EVDIYTVK	483.7608	2	15.55	17.55	27
LDHA	**L-lactate dehydrogenase A chain**	LVIITAGAR	457.2951	2	16.05	18.05	27
	P00338 (LDHA_HUMAN)	FIIPNVVK	465.2946	2	27.24	29.24	27
PTGS1	**Prostaglandin G/H synthase 1**	ILPSVPK	377.2471	2	11.8	13.8	27
	P23219 (PGH1_HUMAN)	VPDASQDDGPAVERPSTEL	661.6482	3	20.46	22.46	27
		LILIGETIK	500.3261	2	27.45	29.45	27
FASN	**Fatty acid synthase**	LLEQGLR	414.7505	2	10.38	12.38	27
	P49327 (FAS_HUMAN)	FPQLDSTSFANSR	735.3546	2	26.48	28.48	27
AGR2	**Anterior gradient protein 2 homolog**	LPQTLSR	407.7427	2	7.16	9.16	27
	O95994 (AGR2_HUMAN)	HLSPDGQYVPR	423.5509	3	9.3	11.3	27
PARK7	**Protein/nucleic acid deglycase DJ**	VTVAGLAGK	408.2529	2	10.47	12.47	27
	Q99497 (PARK7_HUMAN)	DGLILTSR	437.7533	2	17.36	19.36	27
AMACR	**Alpha-methylacyl-CoA racemase**	LQLGPEILQR	583.8482	2	28.39	30.39	27
	Q9UHK6 (AMACR_HUMAN)	LAGHDINYLALSGVLSK	590.9965	3	34.56	36.56	27
SMS	**Spermine synthase**	HSTLDFMLGAK	610.3106	2	23.39	25.39	27
	P52788 (SPSY_HUMAN)	YWPTADGR	483.2274	2	13.74	15.74	27
LDHB	**L-lactate dehydrogenase B chain**	IVVVTAGVR	457.2951	2	13.1	15.1	27
	P07195 (LDHB_HUMAN)	MVVESAYEVIK	634.3338	2	24.62	26.62	27

AMACR, a known marker for PCa, and one of the proteins with the highest fold change and statistical significant different in the shotgun experiments, was included as positive control for the PRM measurements. ANOVA comparison between NAP and PCa at different Gleason Scores indicated a high up-regulation in PCa (p<0.0001) for AMACR, confirming the validity of our PRM set up. We could also confirm the shotgun results of AGR2 and FASN proteins using PRM, in both cases the peak area and the Gleason score correlated well. A summary of the statistical calculations performed for the 18 proteins analysed by PRM is presented in Supplementary Table 5.

To validate the importance of the AA pathway in PCa and its possible application in prognosis, we analysed seven AA pathway proteins by PRM. We included LOX5 as it is involved in the production of eicosanoid-like compounds, and it was not identified by shotgun. Statistical analysis presented in Supplementary Table 5 indicated that LOX5 and TEBP are highly up-regulated in PCa tissue and they are also correlate with the Gleason score. On the other hand, LX15B, HYES, PGH1 were down-regulated (ANOVA, p<0.001) and LKHA4 and FAAH were not different when compared with the Gleason score.

To identify whether selected proteins involved in metabolic reprogramming (Warburg effect) are de-regulated in PCa, we included transitions for another seven proteins in the PRM setup. Interestingly, the ANOVA test indicated that ACSL3, GLSK, LDHA, LDHB and ANM1 proteins are statistically significant (p<0.001). Two proteins involved in polyamine metabolism, SMS and SRM, were not different between NAP and PCa, even though this pathway is known to be heavily deregulated in PCa [10].

### Immunohistochemistry

**Figure 3:**
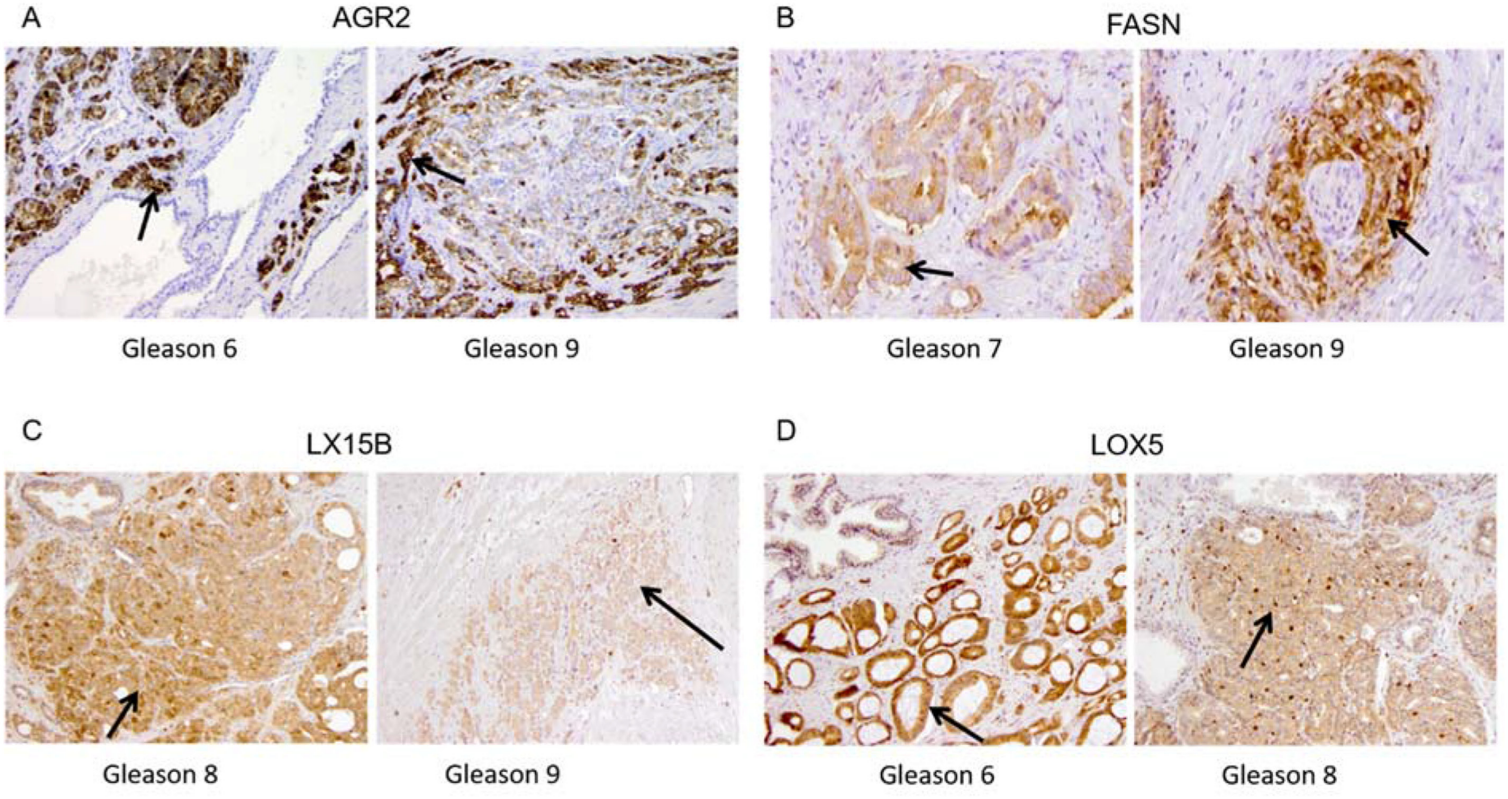
Immunohistochemical staining in PCa tissue for **(A)** Anterior Gradient 2 (AGR2) in Gleason score 6 and Gleason score 9, **(B)** Fatty Acid Synthase (FASN) in Gleason 7 and Gleason 9, **(C)** 15-lipoxygenase-2 (LX15B) in Gleason score 8 and Gleason 9 and **(D)** 5-lipoxygenase (LOX5) Gleason 6 and Gleason 8.

Based on the results of the mass spectrometry experiments we selected four proteins for immunohistochemical (IHC) validation: AGR2, FASN, LOX5 and LX15B. These proteins were selected based on their correlation with the Gleason score and the availability of antibodies. In addition, these antibodies ought to work reliably using formalin-fixed paraffin-embedded (FFPE) tissue. IHC staining of the selected proteins on PCa FFPE tissue sections is shown in Figure 3.

AGR2 showed heterogeneous expression in normal luminal epithelium and PCa. AGR2 staining was strikingly positive in cancer and negative in normal (Figure 3A). Expression of cytoplasmic FASN was negative to weak and rarely moderate in normal prostate luminal epithelium. Expression in PCa was stronger (1+/2+) than in adjacent normal tissue (0/1+) with locally strong expression (3+) in Gleason grade 7 and 9 areas (Figure 3B).

Expression of LX15B was generally moderate to strong (2+/3+) and occurred in both cytoplasm and nucleus of both benign luminal cells and PCa. Normal basal epithelium and atrophic prostate epithelium generally showed lower expression (0/1+). Stromal expression was negative (0) to weak (1+) (Figure 3C).

LOX5 staining was found to be predominantly expressed in the nuclei and cytoplasm of benign basal epithelial cells and atrophic luminal epithelial cells (1+ to 3+). Normal luminal epithelial cells were generally negative (0) or weakly positive (1+). PCa showed enhanced expressions as compared to benign luminal cells varying from weak to strong, but no clear association with the Gleason score was observed (Figure 3D).

### Tissue microarray

To determine whether expression of AGR2, FASN, and LOX5 might correlate with clinical parameters we analysed these proteins in 481 samples from RP patients. Cytoplasmic expression of AGR2 occurred in 84% (404/481) of the patients, with 52% of cores showing strong intensity (3+). 74% (299/404) of the cores exhibited staining in 100% of tumour cells. Strong FASN staining occurred in 86% (399/461) of patients. We did not find any expression of LOX5 in 54% of the cores, and both the cytoplasmic and the nuclear intensities were weak (1+) in most cases. The percentage of positive tumour cells stained for LOX5 was lower than 10% in the 224 positive cores.

An association between the Gleason score (GS) and the percentage of positive tumour cells and intensity of AGR2 was found (p=0.017, and p=0.032, respectively, as described in Table 2A-2B). AGR2 expression occurred more often in patients with lower GS (42% in patients with GS <7 when analysing percentage of tumour cells, and 52.3% when cytoplasm was analysed). FASN expression was higher in GS <7 and GS=7 (49.0% and 34.6% respectively) than in GS>7 (5.4%), but no correlation existed between FASN and PSA, GS or pT stage (Supplementary Table 6).

**Table 2A:** Clinico-pathologic correlations in the PCa-TMA and AGR2 (percentage of positive tumour cells)

	Negative	Positive	Total	*p-value*
PSA at diagnosis				
≤10 ng/ml	88 (21.8%)	264 (65.3%)	352 (87.1%)	0.141
>10 ng/ml	18 (4.4%)	34 (8.5%)	52 (12.9%)	
Total	106 (26.2%)	298 (73.8%)	404	
Gleason score				
<7	44 (10.9%)	170 (42.0%)	214 (52.9%)	0.017
7	51 (12.6%)	112 (27.6%)	163 (40.2%)	
>7	11 (2.7%)	17 (4.2%)	28 (6.9%)	
Total	106 (26.2%)	299 (73.8%)	405	
pT-stage				
pT2	68 (16.8%)	212 (52.3%)	280 (69.1%)	0.121
pT3/4	38 (9.4%)	87 (21.4%)	125 (30.9%)	
Total	106 (26.2%)	299 (73.8%)	405	

Positive = 100% positive cells, Negative = less than 100% positive tumour cells.

**Table 2B:** Intensity of positive tumour cells, Negative = weak or no staining, Positive = strong staining intensity

	Negative	Positive	Total	*p-value*
PSA at diagnosis				
≤10 ng/ml	9 (2.2%)	343 (84.9%)	352 (87.1%)	0.203
>10 ng/ml	3 (0.8%)	49 (12.1%)	52 (12.9%)	
Total	12 (3.0%)	392 (97.0%)	404	
Gleason score				
<7	2 (0.5%)	212 (52.3%)	214 (52.8%)	0.032
7	8 (2.0%)	155 (38.3%)	163 (40.3%)	
>7	2 (0.5%)	26 (6.4%)	28 (6.9%)	
Total	12 (3.0%)	393 (97.0%)	405	
pT-stage				
pT2	9 (2.3%)	271 (66.9%)	280 (69.2%)	0.465
pT3/4	3 (0.7%)	122 (30.1%)	125 (30.8%)	
Total	12 (3.0%)	393 (97.0%)	405	

A correlation between pT stage and cytoplasm intensity of LOX5 was found (p=0.044, in Supplementary Table 7A). No other correlation was found when analysing cytoplasmic intensity, nuclear intensity, or percentage of positive tumour cells for LOX5 (Supplementary Table 7B-7C).

**Figure 4:**
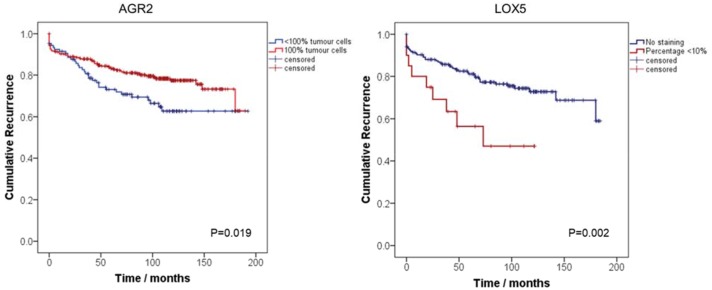
Kaplan-Meier curves assessing the probability of PCa biochemical recurrence after radical prostatectomy by AGR2 and LOX5 Blue lines represent: AGR2: Percentage of tumour cells <100% LOX5: No staining. Red lines represent: AGR2: 100% of positive tumour cells, LOX5: >0% staining of positive tumour cells.

We constructed Kaplan Meier (KM) curves to identify the role of AGR and LOX5 in predicting BCR after surgery. We analysed if the percentage of positive cells of AGR2 100% (positive) or lower than 100% (any negative) was predictive for BCR. We found that a percentage lower than 100% of positive tumour cells (<100%) in AGR2 was predictive for BCR (HR (95% CI) = 0.61 (0.43-0.93)); p=0.02), as described in Table 2 and Figure 4.

For LOX5, we analysed whether the percentage of positive cells was negative (LOX5 0%), or it had any expression (LOX5 > 0%). Expression for LOX5 was characterised by only a small percentage of positive tumour cells, being at maximum 10%. KM curves indicate that low percentage of LOX5 positive tumour cells is a predictor of BCR in comparison with negative staining (0%), in a univariate analysis (HR (95% CI) = 2.53 (1.23-5.22)); p=0.002), as presented in Table 2 and Figure 4.

## DISCUSSION

The Gleason score is an effective indicator of aggressiveness of PCa and therefore an important parameter to determine prognosis. However, more knowledge of which patients will relapse after radical prostatectomy and/or which patients will respond better to a specific treatment is still needed.

AGR2 is a predictor of biochemical recurrence after performing TMA immunostaining in the Evaluation set (Figure 4), confirming previous reports for this protein as biomarker for PCa [13–15]. Bu *et al*., demonstrated that AGR2 is overexpressed in PCa, particularly in low-grade tumours and also in tumour precursor lesions PIN. In addition, high levels of AGR2 transcript were found in urine sediments from PCa patients [16]. Two distinct splice variants of AGR2 in urine exosomes have been identified as effective markers distinguishing NAP and PCa [17]. AGR2 has been reported to be induced by androgens in PCa [18], and its tumorigenic function is associated with cell growth, survival and metastasis, as recently reviewed [19].

Fatty acid synthase (FASN) is known to be a key enzyme in the production of long chain fatty acids from Acetyl-CoA and Malonyl-CoA [20]. Overexpression of this protein in PCa tissue has been reported in cell lines [21], tissue microarrays [22], tissue biopsy cores [23] and exosomes [24]. FASN-normalised intensity was high in PCa in our proteomics dataset and its expression was independently evaluated by immunohistochemistry and a TMA. Although its expression does not predict biochemical recurrence, inhibition of FASN has been proposed as a therapeutic target because of its increased expression and its relation to both cell cycle arrest and apoptosis [25]. Our results reinforce the theory that FASN could be an important target to manipulate the fatty acid and lipid metabolism in cancer and therefore control cancer cell behaviour [26–28].

The AA pathway is a key inflammatory pathway involved in cellular signalling as well as prostate carcinogenesis [29]. Arachidonic acid is stored in cell membranes as a phospholipid, it is released by the action of phospholipase A2-type enzymes, and then metabolised by the action of cyclooxygenases (COX), lipoxygenases (LOX) and P450 cytochromes to produce biologically active eicosanoids [8].

We found that the abundance of lipoxygenase 15 type 2 (LX15B), an enzyme encoded by the gene *ALOX15B*, was lower in PCa than in NAP. Evaluation by immunohistochemistry showed a moderate increased abundance in both cytoplasm and nucleus of normal luminal cells and PCa when compared to normal basal epithelium. These results do not support our hypothesis that the previously reported high serum concentration of HETE metabolites could be explained by an up-regulation of this lipoxygenase-type enzyme [6]. Interestingly, low expression of LOX5 in PCa tissue is slightly higher in PCa compared to NAP, and this expression can be used to evaluate BCR after surgery (Figure 4). In addition, we noticed that expression of upstream enzymes, such as the phospholipases (PA2PA), is higher in PCa when there is an activation of ERG-oncogene (Supplementary Table 4). These results highlight the importance of the AA pathway in PCa, and, particularly, when ERG is activated. However, functional studies need to be performed in order to analyse the link between ERG activation and the de-regulation of enzymes in the AA pathway, as well as the HETE metabolites or other eicosanoid-type fatty acids in the development of PCa.

Association of different enzymes of this family with PCa has recently been described in literature. Patel *et al*. [30], studied the expression of cytosolic phospholipase A_2_ in PCa cells and they reported that increased levels of this enzyme were observed in androgen-insensitive PCa cell lines and they suggested that this enzyme plays a role in cancer cell proliferation and apoptosis. PAFAH (*PLA2G7*) enzyme was identified by Vainio *et al*. in a set of 9783 human tissue samples and it was proposed as a potential drug target specially in ERG positive PCa [31]. Validation studies performed by the same group indicated a correlation between staining intensity for PAFAH and Gleason Score in 50 % of the cases, thus suggesting that both enzymes can be seen as biomarkers for PCa, and the PAFAH inhibition by statins as a therapeutic tool for managing the disease [29].

We found that the protein TEBP (*PTGES3*), was up-regulated in PCa tissue, TEBP protein is involved in eicosanoid signalling as it produces the Prostaglandin E2, involved in inflammation processes. In addition, it is reported to be an enhancer of androgen receptor activity. It is involved in AR binding to chromatin, which is a critical step in AR signalling and PCa development [32, 33]. Further validation is still required, using both quantitative mass spectrometry and immunohistochemistry, to confirm a potential role of this protein in PCa diagnosis and prognosis. In addition, further analysis *in-vitro*, could address the role of the metabolite prostaglandin E, produced by TEBP along the AA pathway in PCa development and progression.

Cancer cells demand energy for proliferation and therefore there might be a metabolic reprograming in the cancer progression process (Warburg effect). By using both shotgun and PRM experiments, we noticed that some metabolic enzymes are de-regulated in PCa (Supplementary Table 5). It is of interest that the expression of lactate dehydrogenase-A protein (LDHA) was significantly (p<0.0001) lower in PCa than in NAP, and it is also de-regulated when ERG is activated (Supplementary Table 4). This result might be associated to previous reports for this protein indicating a key role in PCa oncogenesis [34]. LDHA executes the final step of aerobic glycolysis and has been reported to be involved in tumour progression [35]. It was recently demonstrated that LDHA overexpression is highly linked to local relapse of PCa [36].

In conclusion, the experiments in this study allowed the identification of proteins and pathways associated to PCa. We identified a relationship between proteins in the AA pathway and PCa, and we have shown that expression of LOX5 and AGR2 in tissue predict biochemical recurrence after radical prostatectomy. Further validation studies on independent cohorts using different antibodies are needed to analyse the role of TEBP in PCa progression, as well as their clinical applicability. In addition, functional analyses are still required to fully understand their role in cancer cell proliferation, apoptosis and senescence.

## MATERIALS AND METHODS

### Clinical specimens

### Discovery set

The protein fractions from tissue RNA isolation of 67 samples (33 NAP tissues and 34 PCa tissues) were analysed (MEC-2004-261); PCa samples were previously published (GSE41408) [37], as well as additional cancerous and control samples, accessible via GEO accession number GSE59745 [38]. Clinico-pathologic characteristics of the samples used for proteomics profiling are presented in Supplementary Table 1.

### Tissue Microarray (TMA)- evaluation set

A Tissue Microarray (TMA) was constructed including 481 patients diagnosed with PCa from the European Randomized Study of Screening for PCa (ERSPC) [39–41]. All patients had undergone RP in Erasmus MC between 1987 and 2010, without previous radiation or hormonal therapy. Clinical follow-up was recorded after each control visit at our outpatient clinic, and data were transmitted to a central study database. Post-operative biochemical recurrence (BCR) was defined as an increment of 0.2 ng/mL in serum PSA after two consecutive measurements, with at least three months between measurements. Clinico-pathologic characteristics and follow-up for patients treated by RP are also summarized in Supplementary Table 1.

### Sample preparation

#### Proteomics

The protein interface from tissue RNA isolation with RNA-Bee of 67 PCa tissue samples (33 NAP adjacent tissues and 34 PCa) were kept at -80 °C. For protein digestion, samples were thawed and 50 μL were transferred to a new microcentrifuge tube and precipitated with cold acetone. After spinning down for 10 minutes, the supernatant was removed and the pellet was washed twice with cold acetone. Supernatant was removed and 50 μL 0.1% RapiGest (Waters Corporation, Milford, MA) 50 mM NH_4_HCO_3_ were added to the protein pellet. The protein pellet was dissolved by external sonication for 5 min at 70% amplitude at a maximum temperature of 25 °C (Digital Sonifier model 450, Branson, Danbury, CT). The proteins were reduced with 10 mM dithiothreitol (DTT) at 60 °C for 30 min. After the mixture was cooled down to room temperature, it was alkylated in the dark with 50 mM iodoacetamide at ambient temperature for 30 min, and digested overnight with 8 μL trypsin 0.1 μg/mL (Promega, Madison, WI). To inactivate trypsin and to degrade the RapiGest, 6 μL of 5% TFA was added and samples were incubated for 30 minutes at 37 °C. Samples were centrifuged at maximum speed for 60 minutes at 4 °C and the supernatant was transferred to a new Eppendorf tube. A fraction of 5 μL was then diluted 40 times and subsequently transferred to LC vials for LC-MS analysis.

### Chromatography separation and mass spectrometric analysis

Samples were measured using a nano-LC system (Ultimate 3000, Thermo Fisher Scientific, Amsterdam, the Netherlands) coupled online to Q Exactive plus mass spectrometer (Thermo Fisher Scientific, Bremen, Germany). Chromatographic and mass spectrometry conditions used are described previously [42, 43]. Briefly, 2 μL were injected into the nano-LC after preconcentrating and washing of the sample on a C18 trap column (1 mm×300 μm internal diameter) Thermo Fisher Scientific). Peptides were eluted after loading the sample on to a C18 column (PepMap C18, 75 μm ID × 500 mm, 2 μm particle and 100 Å pore size, Thermo Fisher Scientific) using a linear 90 min gradient (4-25% acetonitrile/H_2_O; 0.1% formic acid) at a flow rate of 250 nL/min. The separation of the peptides was monitored by a UV detector (absorption at 214 nm). Data was collected in data-dependent acquisition mode (DDA). Full scan MS spectra (m/z 400-1600) in profile mode were acquired in the Orbitrap with a resolution of 70,000 after accumulation of an AGC target of 1 × 10^6^ using a maximum fill time of 100 ms. The top 12 peptide signals (charge-state 2^+^ and higher) were isolated (1.6 Da window) and fragmented by HCD (Higher-energy collision, normalized collision energy 28.0) and measured in the Orbitrap with a AGC target of 50,000, a maximum fill time of 60 ms and a resolution of 17,500. Dynamic exclusion was activated; after the first time a precursor was selected for fragmentation it was excluded for a period of 30 seconds using a relative mass window of 10 ppm. Lock mass correction was activated to improve mass accuracy of the survey scan.

Technical replicates of each sample were randomly analysed within the measurement period and no significant changes in the number of identified proteins were observed in time for both the replicates and quality control measurements

### Orbitrap-MS/MS data processing and analysis

Label free Quantitation was performed using MaxQuant software (version 1.5.5.1) [44]. Data was searched against the UniProt-Swiss-Prot 2014-4 database using the Andromeda [45] search engine incorporated in MaxQuant. Cysteine carbamidomethylation was set as fixed modification and methionine oxidation and N-terminal acetylation were set as variable modifications. Peptide and protein identifications were set at a maximum False Discovery rate of 1%. We used the option “match between runs” option to allow matching identifications across measurements and the minimum number of peptides per proteins required for quantitation was set to 2.

### Parallel reaction monitoring (PRM)

PRM was performed on a nano-LC Fusion Orbitrap system. We used similar settings to the above-mentioned DDA measurements on the nano-LC, with the difference of an elution gradient of 60 min. A targeted MS/MS method was developed for 37 peptides, as presented in Table 1. A quadrupole isolation window of 1 m/z units, an AGC target of 2e5 ions, a maximum fill time of 502 ms and an orbitrap resolving power of 240,000 at 200 m/z were used. A fixed HCD normalized collision energy for all peptides of 27 was used, retention times were determined for all peptides, using multiple injections of a tissue sample in which a signal for all 27 peptides was present. Based on the determined retention times a scheduled method was established using a 3 minutes retention time window for each peptide.

### Immunohistochemistry

Tissue slides (5 μm) were mounted on aminoacetyl-silane coated glass slides (Statfrost, Berlin, Germany), deparaffinised in xylene and dehydrated in ethanol. Endogenous peroxidase was blocked by 1% hydrogen peroxide in methanol for 20 min. Samples were pretreated by microwave (700 W) in TRIS-EDTA pH 9.0 or in citrate buffer pH 6.0 for 15 min. The slides were incubated overnight at 4 °C with the following primary antibodies targeting anterior gradient protein 2 (AGR2; 1:100; HPA007912, Sigma); fatty acid synthase (FASN; 1:400; ab22759, Abcam, Cambridge, MA, USA); arachidonate 15-lipoxygenase type B (LX15B 1:2000, ab23691, Abcam, Cambridge, MA, USA), and arachidonate 5-lipoxygenase (LOX5; 1:200 ab169755, Abcam, Cambridge, MA, USA) [41]. Chromogenic visualization was performed with the EnVision DAKO Kit (Dako, Glostrup, Denmark). After counterstaining with haematoxylin, slides were thoroughly washed, dehydrated, cleared in xylene and mounted in malinol (Chroma-Geselschaft, Körgen, Germany).

In the tissue microarray, immunohistochemical staining for AGR2, 5-LOX and FASN was visually examined as described previously [46]. Staining intensity was scored as negative (0; no staining), weak (1+; only visible at high magnification), moderate (2+; visible at low magnification), and strong (3+; striking at low magnification). If there was heterogeneous expression, the strongest intensity was used for further analyses. For AGR2, the percentage of positive tumour cells was counted and used for further analyses. For optimization and validation of all immunohistochemical procedures we used appropriate internal and external controls, and omitted first antibodies to exclude non-specific binding [47].

### Statistics

Protein annotation and statistical testing for differences (two-sided Student's T-test, permutation-based FDR 0.05) in the proteomics shotgun experiments was performed in Perseus [48]. Protein normalised intensities were log2 transformed before testing. The PRM data were analysed using Skyline version 3.5.0.9320 MacCoss Lab Software, Seattle, WA; https://skyline.gs.washington.edu/labkey/project/home/software/Skyline/begin.view), fragment ions for each targeted mass were extracted and peak areas were integrated. Data matrix from PRM experimenst was processed in GraphPad Prism 5 for Windows and R version 3.2.3. One-way Analysis of Variance (ANOVA) allowing multiple comparisons was used to estimate differences among Gleason score groups.

Associations between clinico-pathologic parameters and protein expression in TMA experiments were performed by student *t*-test or chi-squared test. Survival curves were calculated according Kaplan-Meier (KM), and to detect significant survival differences the Log-Rank test was used. Univariate and multivariate Cox regression were used to determine predictive properties of AGR2, LOX5 and FASN for BCR. A two-sided *p*-value of ≤ 0.05 was considered significant. Statistical analysis for the TMA expression was performed in SPSS version 22.

## SUPPLEMENTARY MATERIALS TABLES




