# Supplementary material for: Tissue proteomics outlines AGR2 AND LOX5 as markers for biochemical recurrence of prostate cancer
Source: Oncotarget. 2018 Nov 23;9(92):36444–56. doi: 10.18632/oncotarget.26342 (PMC6284859; doi:10.18632/oncotarget.26342)
Supplement: Supplementary file 1 [file oncotarget-09-36444-s001.pdf]

Tissue proteomics outlines AGR2 AND LOX5 as markers for biochemical recurrence of prostate cancer

SUPPLEMENTARY MATERIALS

Supplementary Table 1: Clinicopathological characteristics and follow-up of patients treated by radical prostatectomy for prostate cancer

See Supplementary File 1

Supplementary Table 2: Overview of identified and differentially expressed proteins in PCa tissue compared to NAP by shotgun proteomics approach

See Supplementary File 1

Supplementary Table 3: List of proteins in the AA pathway according to Sabidó et al. [1]

See Supplementary File 1

Supplementary Table 4: Differentially expressed proteins in PCa tissue when oncogene ERG is activated (ERG positive VS. ERG negative)

See Supplementary File 1

Supplementary Table 5: Statistical comparisson from parallel reaction monitoring (PRM) experimens in PCa tissue

See Supplementary File 1

Supplementary Table 6: Clinico-pathologic correlations in PCa-TMA and FASN (Intensity of positive tumour cells)

See Supplementary File 1

Supplementary Table 7: Clinico-pathologic correlations in the PCa-TMA, LOX-5 A. intensity in the cytoplasm, B. intensity in the nucleus, and C. percentage of positive tumour cells

See Supplementary File 1

REFERENCES

1. Sabidó E, Quehenberger O, Shen Q, Chang CY, Shah I, Armando AM, Andreyev A, Vitek O, Dennis EA, Aebersold R. Targeted proteomics of the eicosanoid biosynthetic pathway completes an integrated genomics-proteomics-metabolomics picture of cellular metabolism. Mol Cell Proteomics. 2012; 11:M111.014746. https://doi.org/10.1074/mcp.M111.014746
